# The Life and Legacy of Samuel Hahnemann: Founder of Homoeopathy and His Medical Philosophy

**DOI:** 10.7759/cureus.70489

**Published:** 2024-09-30

**Authors:** Bhagyashri K Patil, Madhura A Gandhi

**Affiliations:** 1 Central Research Facility, Dr. D. Y. Patil Medical College, Hospital and Research Centre, Dr. D. Y. Patil Vidyapeeth (Deemed to be University), Pune, IND

**Keywords:** founder of homoeopathy, hahnemann, historical vignette, homoeopathy, medical pioneers

## Abstract

Dr. Christian Friedrich Samuel Hahnemann (1755) was the visionary founder of homoeopathy, a system of medicine that emerged amidst the orthodox practices of his time. This biography traces Hahnemann's journey from a curious scholar in Meissen to a pivotal figure in global healthcare. His relentless questioning and experimentation led to the development of homoeopathy, grounded in the principles of "like cures like" and potentization. Hahnemann challenged prevailing medical norms, emphasizing individualized treatment and minimal dosing, significantly influencing modern healthcare. Despite considerable opposition, his innovations established a new medical paradigm that inspires practitioners and patients today. This paper explores Hahnemann's early life, education, homoeopathy's rise, key innovations, and enduring legacy. His commitment to original thinking and perseverance birthed homoeopathy and transformed approaches to medicine, highlighting its holistic focus and individualized care. Hahnemann is recognized as a pioneer in compassionate healthcare.

## Introduction and background

The homoeopathic system of medicine was first introduced by German scientist Dr. Christian Friedrich Samuel Hahnemann, who was born on April 10, 1755; today, we celebrate his birthday as World Homoeopathy Day [1]. He was an original thinker, pioneering experimenter, and meticulous scientist with advanced ideas ahead of his time [2].

During the time of his birth, the method of treating patients was very harsh. Patients endured harsh treatment, including bloodletting, where veins were cut to remove "impure" blood, the use of leeches to suck blood, and vicious purgatives [3]. The medical field lacked direction and scientific principles. His journey from a young scholar in Meissen to the founder of a globally recognized medical system, homoeopathy, underscores his brilliance, perseverance, and unwavering commitment to advancing human health [4].

This biography explores the life and legacy of Dr. Hahnemann, highlighting his early years, pioneering research, and significant impact on medicine. From his formative years in Meissen to his revolutionary work and lasting influence, Hahnemann's story is one of intellectual courage and compassionate innovation, illuminating a path that continues to inspire and heal today.

## Review

Early life

**Figure 1:**
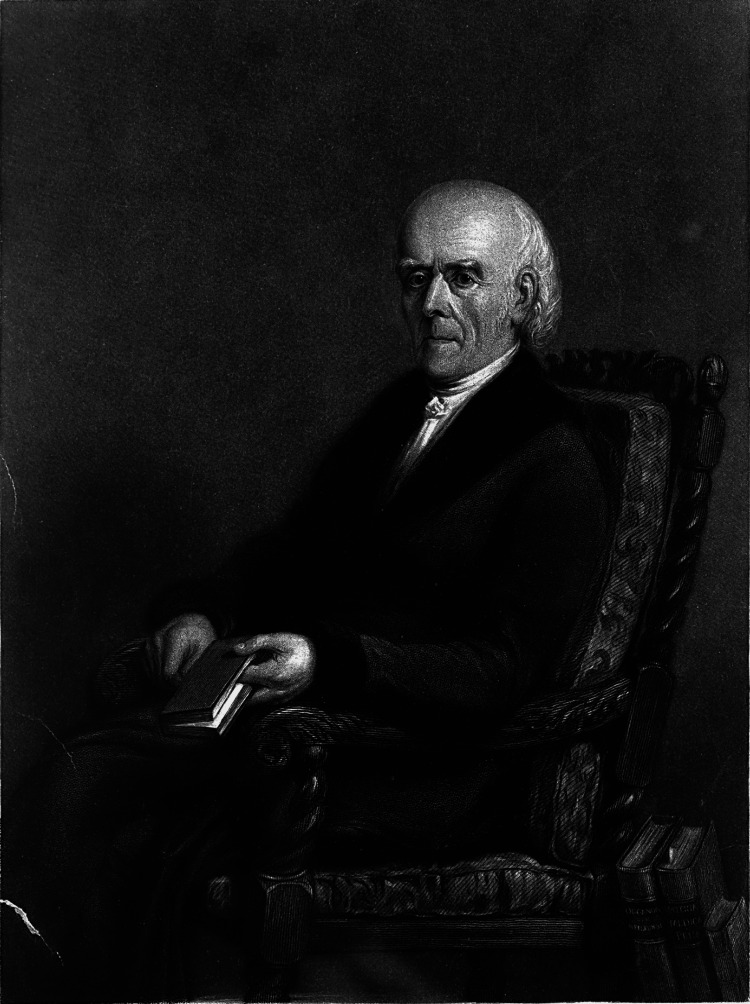
Dr. Christian Friedrich Samuel Hahnemann Source: Wikimedia Commons [6]

Samuel Hahnemann (Figure 1) was born in Meissen as the third child of a porcelain painter [5,6]. His parents quickly taught him to read and write at an early age [5]. His father encouraged intellectual development by having him study alone for hours, which fostered Samuel's habit of original thinking from a young age. His father's principle guided Hahnemann: "Prove all things and hold fast to what is good" [3].

On July 20, 1767, Hahnemann began attending the local school. At 16, he moved to the Prince's School, where he became the favourite student of the principal, Magister Müller. Hahnemann excelled in his studies, mastering several languages and even being commissioned by the principal to teach the basics of Greek to others [4].

Due to his family's financial difficulties, Hahnemann had to leave school and work as a labourer in the porcelain factory. However, he was later re-admitted to the Prince's School with the support of the school's staff. In 1775, his final essay upon leaving the school was on his chosen topic, "The Wonderful Construction of the Human Hand" [5].

Education and personal life

He moved to Leipzig to study medicine, with just 20 thalers and his father's advice to live honestly and without pretence. In 1777, Dr. Hahnemann's thirst for knowledge of medicine inspired him to leave Leipzig and go to Vienna, where he met Dr. Von Quarin at Brothers of Mercy Hospital. Dr. Quarin became a good teacher and friend of Hahnemann. After a few months, Baron von Bruckenthal, the Governor of Transylvania, invited Dr. Hahnemann to Hermannstadt to work as his family doctor and help manage his library and coin collection [7]. He knew German, Greek, Latin, French, Italian, and English. He was also interested in chemistry, botany, literature, natural sciences, and philosophy. He also worked as a translator of books from different languages [3].

Dr. Hahnemann received his MD from Erlangen University on August 10, 1779. The thesis topic was "A Consideration of Etiology and Therapeutics of Spasmodic Affections" [7].

He started his medical career at Hettstadt. After nine months, he left for the Dessau, where he began working on mining technology and chemistry. His fascination with chemistry brought him to Haseler's pharmacy, where he met Henriette Leopoldine Kuchler [2].

In 1781, he worked as a medical officer at Gommern near Magdeburg. At that time, there had been no doctor for a long time in Gommern. On November 17, 1782, he married Henriette Leopoldine Kuchler at Dessau, and their first child, Henrietta, arrived in 1783 [4]. In 1796, he published his first essay in medical observations titled "On the Skin Diseases."

His proficiency in German, Greek, Latin, French, Italian, and English allowed him to access various medical texts, facilitating the exchange of innovative ideas and theories. This ability to translate and communicate across cultures helped him build networks with peers and gain insights from various medical traditions. He was also interested in chemistry, botany, literature, natural sciences, and philosophy. He also worked as a translator of books from different languages [3]. His writings reached broader audiences, supporting and disseminating his homoeopathic principles. In 1810, Dr. Hahnemann published one of the most informative books, "Organon of Medicine," describing concepts of life, health, disease, treatment, cure, etc. [3].

Melanie d’Hervilly was Dr. Hahnemann's second wife. They met in Paris while Hahnemann worked on homoeopathy and seeking better health conditions. Melanie was well-educated. She was 35, while Dr. Hahnemann was around 80 when they married. This age gap was notable, but vital companionship and respect marked their marriage. They married in 1835. Melanie was very supportive of Hahnemann's work and his approach towards medicine [8].

Birth of homoeopathy (the law of similia)

During his translation work, Hahnemann made a significant discovery that would impact millions. While translating Cullen's Materia Medica, he was stunned by a statement: "Cinchona bark (quinine) cures malaria because it is bitter in taste." Hahnemann does not find any logic in that statement [9]. Hence, he thought of experimenting on his own, and he started experimenting on himself by taking four drams twice daily of quinine for a few days. He observed symptoms in himself similar to malaria, such as fever, chills, and exhaustion. After that, he started experimenting with other substances, such as vegetables, chemicals, and toxins to prove that the different substances have different capacities of producing specific symptoms and can cure those symptoms. Hence, the first fundamental principle of homoeopathy, called "the law of similars," was introduced, and another system of medicine, "homoeopathy," was born [10].

Defending homoeopathy against opposition

Hahnemann was busy finding the effects of different medicinal substances on healthy human beings. He experimented with various drugs on himself and his students [3]. They make note of all the minute symptoms. A total of 90 drugs were tested and proven carefully by Hahnemann [3]. The process of drug proving was conducted exclusively on healthy individuals without any clinical abnormalities.

The physicians practising the orthodox system of medicine disagreed with Hahnemann's treatment method [3]. They pressured him to stop his practice or leave the country. Then, he tried to settle in various places; on the other hand, the flow of patients from all over Europe kept coming for treatment.

Another group of physicians practising allopathy appointed Dr. Hering to prove that Hahnemann and homoeopathy are pointless. Hering is interested in medicine and completed his degree from the University of Leipzig, where he was a favourite student of Dr. Henrich Robbi, a renowned surgeon who criticised homoeopathy. C. Baumgartner, who owned a publishing house in Leipzig, wanted a book to disprove homoeopathy and asked Robbi to write it. Robbi was busy with his work and recommended his young assistant, Hering.

Beginning his work in 1821, Hering nearly completed the book by the winter of 1822. However, as he delved into Hahnemann's writings to challenge homoeopathy, he adhered to Hahnemann's advice of repeating experiments before forming conclusions. When he repeated the Cinchona experiment, he found the results aligned with Hahnemann's claims. This further investigation into homoeopathic remedies convinced him of their efficacy [10].

In the winter of 1824, Hering suffered a severe injury to his right forefinger while dissecting a corpse, which led to gangrene. Hahnemann's student, Kunner, suggested Hering for homoeopathic treatment and treated with arsenic album, which completely healed the gangrene. This experience transformed Hering's perspective, and he subsequently became a pupil of Dr. Hahnemann, contributing significantly to the field of homoeopathy [11]. He authored 10 volumes of the "Encyclopedia of Materia Medica" [11].

Seven cardinal principles of homoeopathy

Law of Similia

"Similia Similibus Curentur" is a Greek derivation that means "like cures like." Hahnemann's main idea related to homoeopathy is that symptom similarity is the substance that makes a healthy person sick. This substance is used to help cure similar symptoms in a person who is already ill [12].

Law of Simplex (Single Remedy)

Hahnemann believed that only one medicine should be used at a time. He thought this was important because we only know how each medicine works. If you use more than one medicine, it is hard to know which one is helping or if they might interact in ways we do not understand. Using a single medicine keeps the treatment clear and focused [13].

Law of Minimum

Hahnemann believes that using a minimum remedy dose will have a more therapeutic effect on the patient. The curative effect of the medicine depends not only on the selection of a similar remedy but also on the quantity of the medicine. Since homoeopathic medicines act at a dynamic level, only a minute quantity of the medicine is enough to stimulate the dynamically deranged vital force to bring about the necessary curative change in a patient. This quantitative reduction of the medicinal substance is achieved by the method of potentization, which avoids the unwanted medicinal aggravation caused by crude substances and prevents the chances of organ damage [14].

Doctrine of Drug Proving

According to this doctrine, medicines must be thoroughly tested on healthy humans. Drug proving is a methodical process used to explore the effects of a drug on healthy individuals. Testing on healthy humans is essential due to the subjective and mental symptoms, different effects, and modalities involved.

Subjective and mental symptoms: Animals cannot provide subjective or mental symptoms, which are crucial for understanding the full range of a drug's effects.

Different effects: The response to a drug can vary significantly between humans and animals due to differences in physiology and biochemistry.

Modalities: Drug proving in humans reveals how symptoms change with different conditions, such as time of day, weather, or physical activity. Animals cannot provide this detailed information. Testing on healthy humans provides a more accurate and comprehensive understanding of a drug's effects [15].

Theory of Chronic Disease

Samuel Hahnemann's theory of chronic diseases centres on miasms, deep-seated, inherited, or acquired predispositions that influence susceptibility to illness. Hahnemann identified three primary miasms: psora, associated with deficiency, resulting in conditions such as skin disorders and allergies; sycosis, linked to overgrowth and chronic inflammation, such as warts and fibroids; and syphilis, related to destruction and degeneration, leading to severe conditions such as ulcers and necrosis. Chronic diseases are believed to develop from suppressed acute conditions or due to these underlying miasms. Homoeopathic treatment targets these miasms with specific remedies aimed at restoring the body's vital force and addressing both symptoms and root causes. The holistic approach integrates physical, emotional, and mental aspects to achieve long-term health improvements rather than merely alleviating symptoms [16].

Theory of Vital Force

Homoeopathy posits the existence of an invisible vital force within all human beings. This vital force is responsible for maintaining health, and disturbances caused by various factors lead to illness or disease. Homoeopathy emphasizes the integration of mind, body, and spirit in the healing process. The concept of the vital force is unique to homoeopathy, characterized by its spiritual, autonomous, automatic, dynamic, and non-intelligent qualities. It animates the human body in terms of health and disease [17].

Doctrine of Drug Dynamization (Potentization)

Dr. Hahnemann pioneered the process of potentization by starting with minimal doses of tinctures, such as quinine (*Cinchona officinalis*) and belladonna, then diluting them progressively. To minimize the physiological effects and enhance the remedy's potency, he introduced vigorous shaking, which evolved into a systematic method known as potentization or dynamization.

In 1814, he began vigorously shaking the solution for three minutes. By 1818, he introduced trituration, and in 1821, he standardized the process with 10 strong strokes to the bottle. By 1825, Hahnemann viewed homoeopathic medicines as dynamized when the doses became highly diluted. He continued using 10 strokes in 1837 [18].

Hahnemann defined potentization as transforming a substance's properties through mechanical actions such as trituration and succussion, which reveal latent dynamic powers hidden in the substance. The process involves shaking after each dilution to maintain the remedy's activity. Photons are thought to play a role in this transmission of information.

Hahnemann emphasized that precise, vigorous shaking or trituration was crucial for creating effective potencies. He believed a few ineffective strokes produced only diluted solutions, whereas exact, repeated strokes produced potent remedies [18].

Death of a pioneer

**Figure 2:**
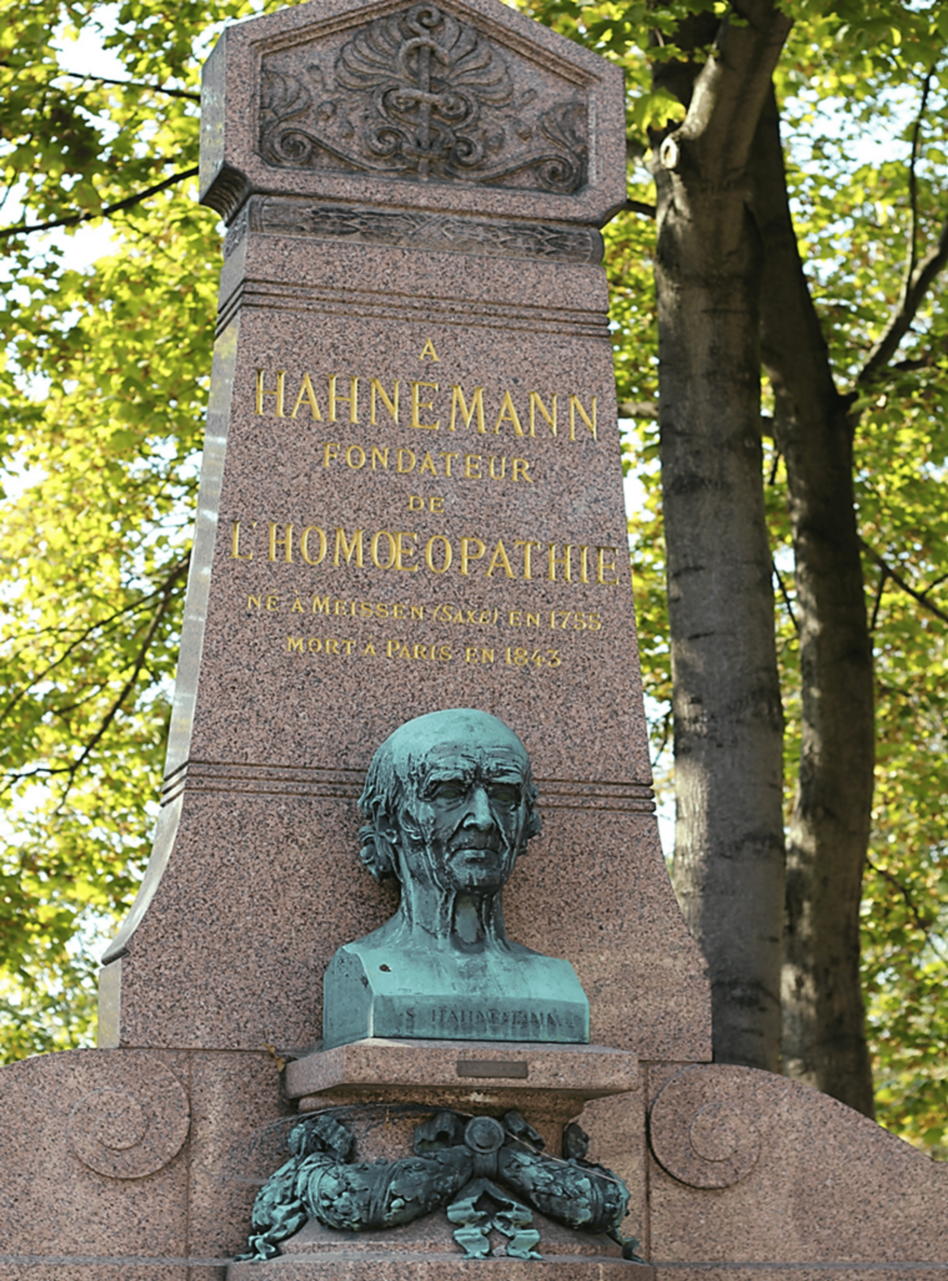
Tomb of Samuel Hahnemann in Paris Source: Wikimedia Commons [20]

Dr. Hahnemann died in Paris, France, on July 2, 1843. In his final days, he was surrounded by his loved ones. He passed away at the age of 89. There was no public funeral for this renowned figure. His body was preserved by Ganal and transported to the Montmartre cemetery on a rainy morning, July 11, 1843 (Figure 2). His wife, daughter, her son, and a few servants attended the final burial ritual [19].

A month after Dr. Hahnemann's death, the Central Society of German Homoeopathists met in Dresden and decided to create a monument to honour their beloved leader. His statue, designed by Steinhauser and cast in bronze in Rome, was revealed during a gratitude ceremony on August 10, 1851 [19].

Dr. Hahnemann's life journey is both challenging and inspiring. He was an actual physician and a revered figure. He remains a gem of both past and modern times. He is Dr. Samuel Hahnemann, the founder of homoeopathy!

## Conclusions

In conclusion, Dr. Samuel Hahnemann's legacy underscores the importance of original thinking and persistence in medicine. His creation of homoeopathy transformed healthcare by emphasizing individualized treatment and the body's natural healing ability. Despite facing challenges, his holistic approach remains relevant today, inspiring many in alternative medicine. Homoeopathy has influenced modern practices by promoting personalized care, increasing interest in integrative therapies, and fostering patient empowerment. Hahnemann's principles of patient-centred care continue to shape contemporary integrative practices, ensuring that his vision for compassionate healing endures in modern medicine.
